# Impact of Attrition, Intercellular Shear in Dry Eye Disease: When Cells are Challenged and Neurons are Triggered

**DOI:** 10.3390/ijms21124333

**Published:** 2020-06-18

**Authors:** Gysbert-Botho van Setten

**Affiliations:** 1 Department of Clinical Neuroscience (CNS), Karolinska Institutet, 11282 Stockholm, Sweden; gysbert-botho.vansetten@sll.se; Tel.: +46-8-672-3298; 2 St Eriks Eye Hospital, 11282 Stockholm, Sweden

**Keywords:** ocular surface, ophthalmology, attrition, corneal epithelium, dry eye, pain, inflammation

## Abstract

The mechanical component in the pathophysiology of dry eye disease (DED) deserves attention as an important factor. The lubrication deficit induced impaired mechano-transduction of lid pressure to the ocular surfaces may lead to the dysregulation of homeostasis in the epithelium, with sensations of pain and secondary inflammation. Ocular pain is possibly the first sign of attrition and may occur in the absence of visible epithelial damage. Attrition is a process which involves the constant or repeated challenge of ocular surface tissues by mechanical shear forces; it is enhanced by the thinning of corneal epithelium in severe DED. As a highly dynamic process leading to pain and neurogenic inflammation, the identification of the impact of attrition and its potential pathogenic role could add a new perspective to the current more tear film-oriented models of ocular surface disease. Treatment of DED addressing lubrication deficiencies and inflammation should also consider the decrease of attrition in order to stimulate epithelial recovery and neural regeneration. The importance of hyaluronic acid, its molecular characteristics, the extracellular matrix and autoregulative mechanisms in this process is outlined. The identification of the attrition and recognition of its impact in dry eye pathophysiology could contribute to a better understanding of the disease and optimized treatment regimens.

## 1. Introduction

Attrition is defined as a decrease or reduction in numbers, size, or strength: a process of making something weaker by repeatedly attacking or exerting processes leading to weakening of resistance. In ophthalmology, attrition has been used as a term for the description of a process involving the constant or repeated challenge of ocular surface tissues by mechanical forces in association with glaucoma surgery [1]. In medicine, the term attrition has been used by dentists in order to describe the effects of bruxism (a specific form of tooth wear) [2]. In orthopedics it is applied to discuss wear resistance [3]. In technical and mechanical sciences, attrition is a summarizing term for processes involving particle breakage [4]. As a summarizing term of constant wear and challenge, the term attrition has been even used in psychology as one of the processes of challenges potentially leading to a burnout [5]. In ophthalmology again, the term attrition has been used in association with structural abnormalities, such as nerves in neurotrophic keratopathy [6]. In the presented model, attrition summarizes all the effects of mechanical forces constantly and repeatedly challenging tissues at the ocular surfaces with friction, stretching and compression. The ability for, and adaptability to deliver mechano-transduction are key elements of attrition. Attrition is herein introduced as a driving force in dry eye pathophysiology, in order to better understand pathological mechanisms in epithelial tissues. Attrition affects the entire cell structure underneath the immediate surfaces suffering from lubrication deficiencies, especially in preclinical stages. Attrition is an initially invisible process leading to initial sensations of pain and irritation. Pain and pain sensations precede visible tissue alteration and could offer an explanation for the mystery of the dissociation of signs and symptoms. This dissociation is one of the key issues in the pathophysiology of dry eye and ocular surface disease. Already, at preclinical stages, impaired function and composition of the extra cellular matrix (ECM) [7] (with fibronectin and hyaluronic acid (HA)) in areas with epithelial thinning could lead to the enhanced intercellular shear and stress. Direct effects such as corneal epithelial erosions and mechanical tissue defects, as well as indirect effects such as cellular reactions, inflammation, and changes in tissue structure, can determine the extent and characteristics of ocular surface reaction and the occurrence of ocular pain, i.e., the intensity of dry eye disease (DED). The concept of attrition allows one to reevaluate the model of the vicious circle of DED [8] and its components.

## 2. Views and News

### 2.1. Characterizing Attrition

**Figure 1:**
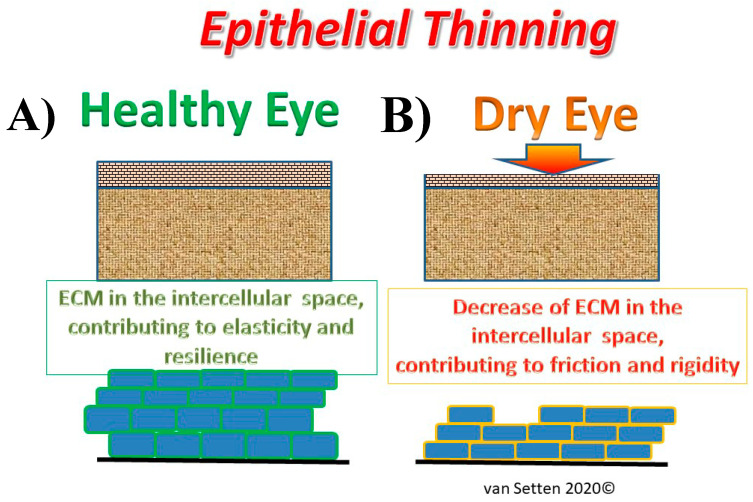
Illustration of the effects of epithelial thinning changing the extra cellular matrix in the intercellular space from a physiological condition (**A**), where the extra cellular matrix (ECM) contributes to elasticity and resilience of the tissue to an impaired condition in DED (**B**) with thinning of the ECM, leading to a compressed intercellular space, contributing to enhanced friction and rigidity.

**Figure 2:**
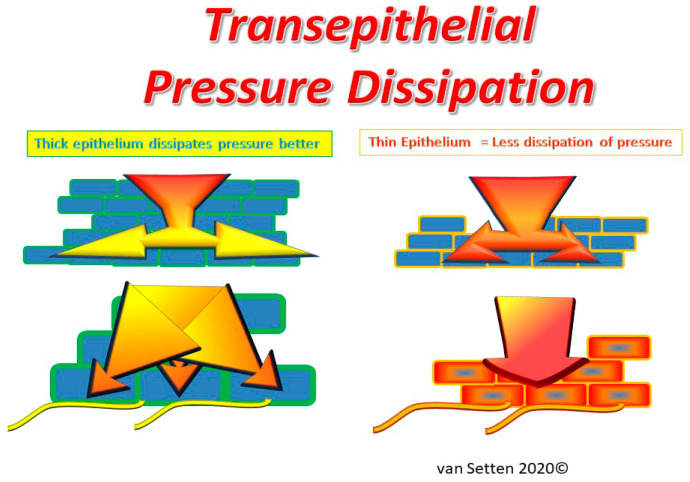
Illustration of the effects of epithelial thinning on transepithelial pressure dissipation, where under physiological conditions, the multilayered normal epithelium dissipates pressure better than a compressed and thinned epithelium with less capacity for dissipation of pressure. The mechano-transduction of any pressure is much more directly directed to the basal cell layer in a thinned epithelium and hence, more likely to cause secondary effects, such as inflammation and pain.

For the ocular surface of the eye, the main exterior force is exerted by the eye lid [9]. Pressure at any surface transfers the main load to the basis of the tissue, which may be a membrane, a basal cell layer or any other organic scaffold. Various parameters influence the effects of the resulting load factor on the underlying tissue. Similar to architecture, the load dissipation in living tissues also plays a major role, depending on load factors, elasticity and the resilience of the tissues. When the pressure of the weight of material (gravity) or mechanical forces (pressure) is exerted onto the foundations of a building, these foundations must be able to cope with these forces. In the same way, the basal layer of a tissue, as well as the structures above the basal layer, must be able to homogenously dissipate and absorb such forces, additional to the ability to tolerate—without damage—vibrations, violent shaking or other lateral or omnidirectional forces. In a building made of bricks, the load on each brick at the basement is inversely proportional to the number of layers of bricks forming the fundament, its geometry and the area that the weight of the building is dissipated onto. In general, the art of architecture does aim to dissipate force, i.e., it spreads out over a greater area, so that no one spot has to bear the brunt of the concentrated force. Or, if intended to carry concentrated forces, it is also constructed to do so. At the epithelium, the pressure of the lid is conveyed through the epithelium to the basement membrane and the underlying tissue. Any thinning of the epithelium and decrease of layers leads to a decrease of dissipation of pressure and an increase in structural stress on each of the cells (Figure 1 and Figure 2). Such thinning of the corneal epithelial thickness occurs in DED [10,11] together with a decrease in cell density [12]. Thinning of corneal epithelium has been observed as a side effect of prolonged treatment of the ocular surface with prednisolone [13] and occasionally after photorefractive keratectomy [14].

Pressure on two layers which pushes them together, such as the lid to the surface, results in friction. This force aggravates any lateral movement of the contacting surfaces of these layers. However, such movement is constantly induced by the movement of the lid, exerting friction on both surfaces and underlying cells, resulting in shear. Such shear forces may influence migration and cell turnover, cause damage to the corneal epithelial cells [15,16] and even result in lid wiper epitheliopathy on the surface of the lid [17,18]. Sufficient lubrication is essential for normal elastohydrodynamics of the eyelid wiper [19], reducing shear forces at the surface. This lubrication is again dependent on the kinetics of tear dynamics [20,21]. The lubrication capacity of tears is determined by their composition and, not least, their quantity. Desiccation causes stress at the ocular surface [22,23]. Besides such lubrication deficit-induced desiccation stress, there is shear stress as another major force affecting the ocular surfaces. Only recently has the higher pressure of the lids onto the ocular surface become associated with a higher incidence of DED and ocular surface damage [24]. Any impairment of the lubrication capacity, such as in DED, increases the shear stress by decreasing the viscoelastic separation between the lid to and ocular surface, leading to friction [25]. This friction is naturally enhanced by the increased tectonic exposure of the ocular surface, such as is suggested for the result of glaucoma surgery [1]. In the presence of local surface elevations, otherwise normal moving forces of the lid create enhanced attrition, summarizing all the effects of pressure and friction [1]. Moreover, minor, local protrusions of the ocular surface decreasing viscoelastic separation between lid to and ocular surface, leading to enhanced friction, such as in the anatomical dry eye [26], can lead to very specific and localized pain sensations. Therefore, such pain can be perceived as far more disturbing and alerting, as it generally occurs in a no-pain condition and herein gains full attention of the body and mind [27]. This pain perception is a primary effect of attrition. Pain could also result from the inflammation as part of a chronic leucocytic inflammatory reaction (CLIRS) [28]. Such triggered nociception (independent if occurring repeatedly or continuously) could be maintained and enhanced by attrition. It is important to point out that, in any individual, central pain amplification may complicate nociceptive or neuropathic pain [27]. Hence, ocular pain management is very important in the treatment of dry eye and ocular surface disorders [29] already, and especially in the initial stages. 

The cornea is the most richly innervated tissue of the eye, with the cornea being the most densely innervated tissue of the human body, whereas the conjunctival has only modest innervation. The principles and details of the corneal innervation have been extensively investigated [30,31,32]. The path of the nerves through the epithelium and their anatomical alteration to finally naked axon cylinders [33,34] make them highly susceptible to mechanical, thermal and osmotic stimulation [28,35]. Although the naked pain nerve fibers are, to a large extent, surrounded either by Schwann cells and/or epithelium cells, parts of the nerve membrane are in direct contact with extracellular matrix, respectively, fluid [36,37].

The neurobiology of the ocular surface and pain has recently been extensively reviewed [35]. Provided that sensory nerves are present and functional, attrition as a result of continuous enhanced friction and its inflammatory response to it, creates sensations and the feeling of pain. These neurogenic sensations do not correlate initially with clinically visible damage at the tissue structure (dissociation of signs and symptoms), as they, initially, resulting from more occasional and subclinical cellular stress (acute leucocytic irritative response syndrome, ALIRS) [28], cannot lead to detectable tissue alterations. Although the neurogenic pain may be intense, the anatomical correlate is still missing. With the progress of tear fluid insufficiency to tear film deficiency, corneal epithelial thickness is decreased in dry eye. Such thinning of various epithelial layers (basal cells show less changes than the wing cell at the surface) [12], together with the thinning of the tear film in patients with DED [38], progressively reduces an essential effective stress buffer from the epithelial surface. High osmolarity, having been associated with severe DED, does also contribute to a decrease of corneal epithelial thickness [39]. Significant variations of osmolarity, such as suggested in the model of osmokinetics in DED [28], would lead to altered variation of pain sensations during the day. Amongst others, substance –P immunoreactive nerves in the corneal epithelium [40] can be activated by the resulting mechanical stimulation and cause pain and discomfort. 

The shear stress, as it occurs at the ocular surface already normally during blinking, is known to affect corneal epithelial cell morphology and the expression of cell junctions [41]. The effects of attrition at the surface are enhanced in DED. In DED the functional deficiency of the lubrication medium, i.e. the tear film at the interface [42], the impaired “coating” of the surfaces with its glycocalyx [43] such as mucins [44,45,46] and the altered surface structure (microvilli, cell turgor etc.) is very important. Deficits in ocular surface lubrication which cause an increase in attrition are further enhanced by local imperfections at the surface, leading to premature break up [47], possibly identifying disease groups of its own [26,48], most recently summarized as “decreased wettability dry eye” [49]. It is at these locations that the first signs of ocular discomfort, as an equivalent of neurogenic irritation, may be perceived. 

Naturally, the first line of treatment of lubrication deficits includes tear fluid substitutes which address specific deficiencies and varies during the stages of the disease [50,51]. Amongst the substances best known to alleviate shear forces at the interfaces are glycosaminoglycans (GAGs), of which HA has been used already in the 80-ies topically on the ocular surface [52] and is now very common. Various HA molecules, as specific attrition influencers/attenuators, have been intensively investigated. It has been found that size, as well as concentration of the HA molecule applied, matters. The specific capacity to reduce shear and alleviate attrition is dependent on the physiochemical properties of the HA and the concentration of HA, and varies with the molecular weight (MW) of the molecule. The ability of HA solutions to reduce shear is dependent on the bulk viscosity (zero shear viscosity) of the hyaluronate solutions used which, in turn, strongly depend both on concentration and MW [53,54].

**Figure 3:**
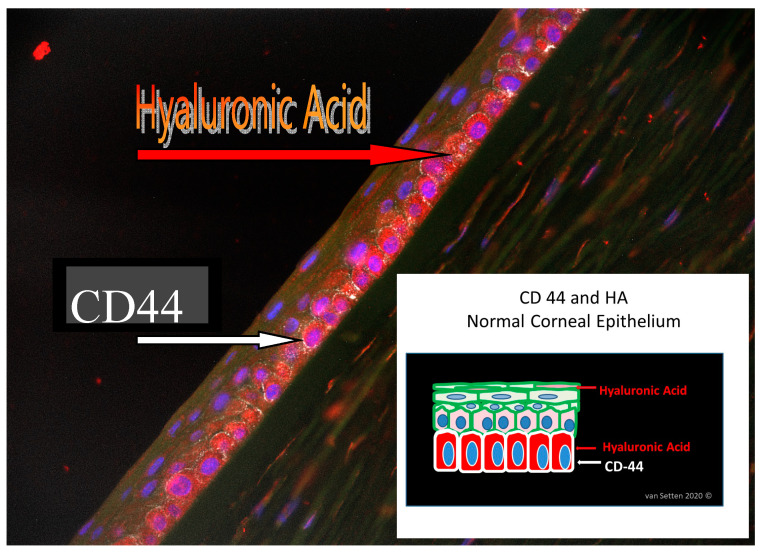
Histology picture identifying HA and CD 44 in human corneal epithelium. The schematic drawing illustrates the localization of HA (stained in red) within the cells and in the ECM even in the wing-cell layer, whereas CD44 (stained in white) is more concentrated in the basal cell layer.

The surfaces covered by the lubricating medium have to adjust to the shear forces they are exposed to. The resulting effects of attrition are directly transferred to the epithelium of the lid and the eye. Here, the abilities of the mechanotransduction of shear forces in the epithelium determine the reactions in the tissue and the nociceptive result of attrition. Mechanotransduction is again influenced by the flexibility of extracellular matrix homeostasis [55]. The extracellular matrix (ECM) contains a plethora of substances, the exact composition varies of which is situation dependent. ECM composition is influenced by ECM turnover, which is influenced and altered amongst other with help of metalloprotease enzymes in response to mechanical loading [56]. Certain ECM molecules may be regulated in response to the type, intensity, and duration of the mechanical stress [57]. Hence, different proteins can contribute to matrices in different situations, which defines the matrisome [7]. Glycoproteins such as HA are a major component of ECM. HA is already normally expressed within the corneal epithelium [58] and heavily involved in wound healing [59]. HA co-localizes with its receptor, transmembrane protein and cluster of differentiation 44 (CD44) [60] (Figure 3), to which it can bind multivalently [61], in particular in its high MW form, emphasizing its important regulative role [62]. CD44 is the main HA receptor [60] and is found as a transmembrane glycoprotein in a wide variety of cell types, including leukocytes, fibroblasts, keratinocytes, and epithelial cells [63]. CD44 also integrates processes in the extracellular matrix (ECM) with the signals of growth factors and cytokines [64]. Expression of CD44 correlates with epithelial regeneration, and is involved in cell-cell interactions, affecting the adhesive strength of the epithelial sheet to the basement membrane [65]. Its expression in proximity to HA suggests regulative interaction during cell migration and re-epithelialization. In the ECM, HA is one of the main components [7], links together as macromolecular aggregates and regulates cell behavior like cell adhesion, motility, growth and differentiation [66,67]. Besides a plethora of functions, hyaluronan is an important space filling molecule [68]. 

Dry eye associated epithelial thinning is accompanied by an altered ECM. The associated decrease of HA as part of the ECM in the intercellular space can lead to an increase of epithelial rigidity and a decrease of the epithelial resilience (resistance to compression), just as has been shown earlier of cartilage or vitreous [69]. This may lead to increased intercellular shear, as equivalent to intercellular friction. 

**Figure 4:**
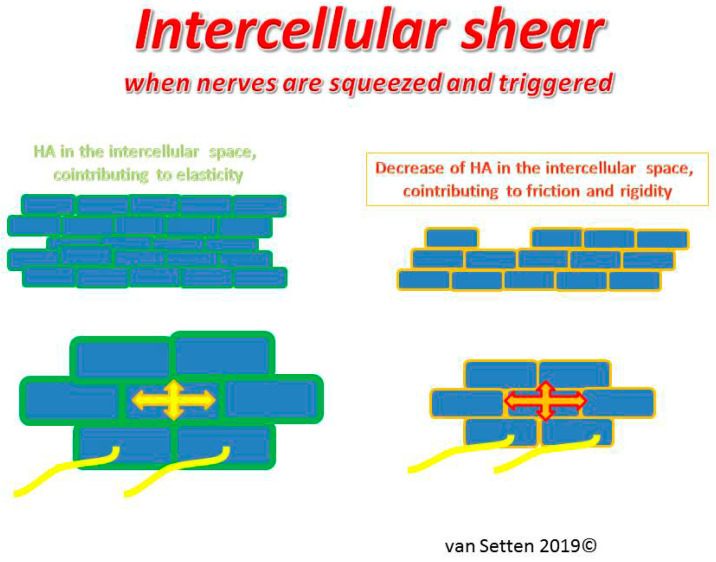
Illustrates the capacity and ease of cell movement and resilience in the normal epithelium in response to pressure in the presence of normal ECM.

**Figure 5:**
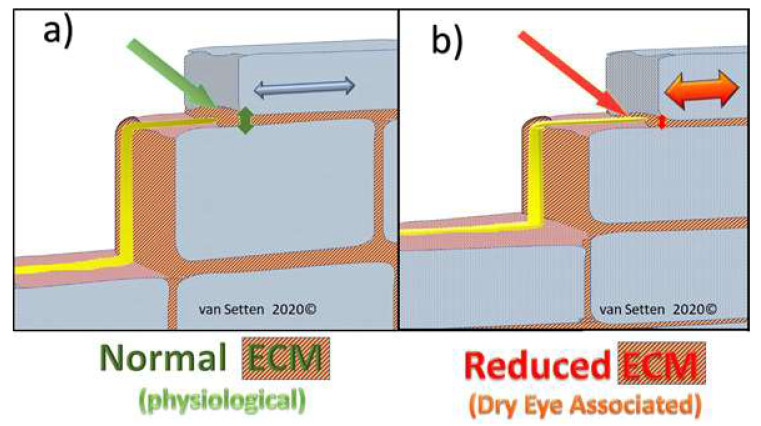
(**a**) Shows, schematically, in a model, how nociceptive nerves in the epithelium pass through the intercellular space, which is maintained with good elasticity and resilience. With decreased ECM, Figure 5 (**b**), intercellular spacing is reduced and mechano-transduction without normal dissipation could lead to enhanced shear and friction, squeezing the axons causing them to fire by every movement of the eye or lid, creating sensation of pain and discomfort.

Higher pressure and enhanced friction in the intercellular space (Figure 4), as well as the enhanced rigidity of cellular membranes in which the nerve axons are embedded in, comes with a more crude and direct mechano-transduction of any shear force exerted upon the ocular surface (contact lenses as well as the lid) to the nerves. Under these conditions, in the preclinical dry eye, the enhanced intercellular interepithelial shear is possibly the anatomical equivalent and reason for discomfort and pain experienced by patients with DED, as nociceptive nerves running through the epithelium are squeezed and triggered by every movement of the eye or lid (Figure 5a,b).

This continuing enhanced mechanical stimulation could, over time, provoke the initiation of chronic inflammatory processes that could facilitate or trigger the vicious circle of DED [8]. In the current model of DED, the ocular surface or epithelial stress is mainly focused on the concept of desiccating and osmotic stress [70], representing a more fluidistic causal model. Although details of the key parameters of the vicious model may regionally vary, emphasizing either osmolarity [71] or tear film break up time [72], these models are tear film focused. There is a current consensus that the disease has a main feature as a self-perpetuating inflammatory disease [73], in which hyperosmolarity, i.e. osmolarity above a threshold value, has been considered a key actor [71]. To this rather static model, recently a dynamic aspect been added, introducing the concept of osmokinetics [28], emphasizing the importance of diurnal variations of osmolarity and identifying its key parameters [74]. In the presented approach, the consideration of attrition as a driving force could add another perspective to the understanding and treatment of DED, adding the importance of epithelial thinning for attrition to the current models [75]. In the area of increased intercellular shear between the cells, the decreased cell mobility and impaired mechano-transduction could become key triggers of ocular surface deterioration. Cellular stress resulting from intercellular shear can be sensed by amongst others by cell membrane receptors [76]. Such sensitivity to mechanotransduction or simply the mechanosensitivity of cells describes the ability of cells to react to environmental stimuli [77]. The basic mechanisms of such mechanosensation without nerval engagement has been reviewed recently [78]. In difference to pain and nerval triggers caused by mechanical forces, mechanosensations are essentially pure protein-protein interactions or enzymatic activities of mechanosensors that respond to external forces such as attrition. One of the principles has been suggested to consist of specific molecules that undergo stretching resulting in conformational changes [78], triggering secondary events. Naturally, these secondary events could reflect on the ECM which, in turn, could lead to neuronal effects. 

The irritation of nerves by constantly enhanced shear in the intercellular space may lead to neurogenic inflammation. This, especially as in the cornea polymodal nociceptors in response to maintained mechanical provocation, may produce a sustained discharge, having a mechanical threshold slightly lower than pure mechano-nociceptors [79,80]. Chronic pain, caused by constant attrition, can trigger inflammation. Any alteration of the ECM could reflect on the metabolism of HA with in this space. Here, on one hand, the production of HA is influenced by inflammation. Hyaluronan itself, on the other hand, is actively engaged in the regulation of inflammation in disease processes and tissue repair [81]. Here, molecular size matters. Especially, low MW HA causes and maintains inflammation [82], emphasizing that hyaluronidases, and turnover of ECM, are of highest importance. On the other hand, high MW HA, such as the molecules of 1300 kDa HA, inhibits induced IL-1β, IL-6, IL-8, IL-4 and IL-10 production in a dose-dependent manner in fibroblasts [83] and displays anti-inflammatory and immunosuppressive properties [84]. The constant balance of synthesis and degradation of HA and its microenvironmental regulation is complex [85], but suggests a constant simultaneous presence of higher and lower MW HA in the ECM. In skin, the observed change from high molecular weight (HMW) HA to low molecular weight (LMW) HA ( for example during wound healing) illustrates the changes of the HA effect from protecting the skin (as HMW-HA) to irritating the skin and causing inflammation as LMW-HA [86]. This could be similar at the ocular surface. This also implies the constant self-regulating presence of inflammation provoking and silencing mechanisms, suggesting that a very moderate, subclinical level of inflammation in the ocular surface epithelium could be normal. The idea of a constant subclinical inflammation in mechanically stimulated epithelia has been recently discussed in context with the recent discovery of tumor necrosis factor-inducible gene 6 protein (TSG-6) in normal corneal epithelium [87]. TSG-6 contains a hyaluronan-binding LINK domain and thus, is a member of the hyaluronan-binding protein family, also called hyaladherins [88,89] and is closely related to CD44 [90]. Its presence in human corneal epithelium is very important, as TSG-6, a multifunctional protein [91], has anti-inflammatory effects and appears to be capable of downregulating inflammatory response [92]. The external application of TSG-6 in the treatment of dry eye has shown promising effects [13], as well as the application of immunomodulators such as ICAM-1 inhibitors [93,94,95,96,97] and Cyclosporin-A [98,99,100,101,102].

In the presence of inflammation, the perception of intercellular stress may augment the neurological sensation from discomfort to pain induced by attrition. Possibly, the so called idiopathic pain in DED is one of the hallmarks of DED and part of the current enigma of dissociation of signs and symptoms in this disease, [103,104,105,106], especially in moderate and mild forms. However, there appears to be significant correlations between ocular pain metrics and dry eye symptom severity scores [107]. Pain and discomfort is for patients a tough reality and a difficulty for the ophthalmologist. Here, the importance of treating inflammation has been emphasized [108]. The nature of pain as nociceptive or neuropathic [79] is often difficult to identify, as components of both may mix or change over time. Here, corneal confocal microscopy offers the ability to quantify the severity of nerve fiber damage in relation to the severity of neuropathic pain [109]. Furthermore, Ross and colleagues described the clinical and in vivo confocal microscopy (IVCM) features of neuropathic corneal pain (NCP) without clinically visible signs, suggesting that peripheral NCP, which responds to topical anaesthesia, and central NCP, which does not, are separate entities [110]. The sensations of DED, such as pain and itching, are associated with inflammation and the activation of specific receptors, such as transient receptor potential vanilloid 1 (TRPV1) [111]. TRPV1 channel activation can again be specifically inhibited by HA [112,113], which may have an essential nociprotective effect. Additionally, HA also reduces toxic effects by blocking Ca2+ influx through TRPV1 channels, preventing Ca2+ overload, leading to nerve cell protection [114]. These specific effects of HA would significantly postpone or alleviate the possible activation of the inflammatory key components of the vicious circle of DED. A decrease of inflammation would normalize local pH which is elevated in the presence of inflammatory cells. Notably, the pH controls the physiological function of TSG-6, with a low affinity for HA at neutral pH, but with increased affinity as the pH falls below pH 7 [115]. The histologically shown simultaneous presence of TSG-6 and HA in human corneal epithelium [87] could hence also reflect a functional interaction. Emphasizing the importance of attrition in dry eye pathology, TSG-6, as mechanical inductible protein [116], may play an active role in the physiological balance of the epithelium. Whether, in turn, enhanced attrition, due to impaired mechano-transduction in dry eye, results in an enhanced TSG-6 expression remains to be investigated. This would be very interesting, as TSG-6 protein forms a stable complex with components of the serine protease inhibitor, inter-alpha-inhibitor (I alpha I) and potentiates the inhibitory effect of l alpha l on the protease activity of plasmin [117], decreasing the cleavage of, amongst others, fibronectin. 

### 2.2. Epithelial Lesions—The Effect of Attrition

Epithelial lesions are the most significant clinical effects of excessive attrition. In the healing events, ECM components such as plasmin and fibronectins are engaged [59,118]. Healing under dry eye conditions with enhanced attrition and in the presence of inflammation is a challenging task for the epithelium. Although the epithelial abnormalities in dry eye disease are initially minor, visible punctate staining of the cornea serves as parameter for estimating the intensity of the disease [119]. The staining with fluoresceine indicates amongst others the presence of epithelial lesions, but its absence cannot exclude the presence of the other pathophysiological equivalent of corneal damage. Additionally, in dry eyes, the interpretation might differ, as the fluorescein staining of the ocular surface is influenced and correlated to the eyelid pressure [120]. However, representing a significant finding in the diagnosis of severe DED [51], epithelial lesions indicate the need for an adequate therapy that provides improved lubrication and support of wound healing events. In the presence of dry eye enhanced shear forces, attrition is augmented by surface imperfections and irregularities, maximizing the lubrication requirements. Even minor irregularities, such as after the use of laser or cataract surgery, can expose the insufficiency of lubrication, even in only borderline dry eyes [26]. In any dry eye condition, the therapeutic rheological optimization is mandatory for treatment success. Here, the rheological, i.e., physicochemical properties, vary a lot and have been reviewed recently for a plethora of tear film substitutes [121]. The lubrication challenge is amplified by the severity of dry eye. Although the positive effect of HA in the treatment of severe forms of dry eye is well recognized and is increasingly reported [122,123], in very severe cases, HA alone, or in combinations, is not sufficient. Therefore, in an environment of elevated attrition, the use of human serum as a treatment for severe dry eyes is well established [124,125]. However, the preparation, storage and transport of autologous serum has its logistic, ethical and legal, as well as regulative, challenges. Therefore is the observation that autologous serum eye drops can, at least temporarily, successfully be replaced by HMW HA eye drops [126] clinically important and very promising. Serum, on the other hand, is used as a diagnostic medium as it may reflect general disease [127]. Hence, pathological conditions of the patient could also alter the blood composition and possibly result in a pathological composition of the serum. When this serum is then applied to the ocular surface, its effects may not be the same as from normal, healthy individuals. The hyper-viscosity occasionally observed in the presence of Sjögren’s syndrome [128] can lead to a hyper-viscous serum that might have impaired lubrication capacity, with a reduced ability to alleviate attrition. Hence, autologous serum is possibly not always an equally efficient tear fluid substitute as standardized pooled serum. 

### 2.3. Reducing the Impact of Attrition: Dry Eye Therapy

Attrition can be therapeutically approached in three ways. First, in addressing the nature of attrition resulting from shear between two surfaces. Therefore, lubrification should be considered as the primary intent of the treatment. The decreased viscosity of tears in patients with qualitative dry eye [129] certainly may contribute to increased friction at the surface, and augmented attrition. Attrition would also be enhanced if the main cause of dry eye were the instability of the tear film, as suggested earlier [130]. Enhanced mechanical forces on an anatomically weakened epithelium will increase the cell stress and the secondary inflammation. Hence, the specific rheological abilities of the lubricants applied have to be considered carefully [131], such as assessing flow properties (fluidity) under different conditions. Additionally, the viscosity of the lubricants has to be evaluated in relation to the shear rate, in order to predict the performance of the lubricant, i.e., its ability to decrease attrition. Depending on the severity of DED, the intensity of inflammation, as well as the location of inflammation, anti-inflammatory drugs such as low-dose corticosteroids in short time applications or immunomodulators such as ICAM-1-inhibitors and cyclosporine, will help to decrease the acute or chronic inflammation [51]. This amelioration of the interface itself, by increasing the lubrication efficiency and decreasing the immediate friction, usually decreases attrition. Additionally, sodium hyaluronate might have a direct role in the control of ocular surface inflammation [132]. However, the real improvement of the situation and the decrease of attrition requires the reconstitution of the intercellular buffer function of the intercellular space, i.e. the ECM and the reorganization of the epithelial structure. This anatomical and physiological reconstitution will follow the visible improvement of corneal staining with some delay. This is due to the characteristics of the epithelium as one of the fastest healing tissues in the human body. The swift healing of the epithelial surface horizontally will be much faster than the recovery of corneal thickness (vertical healing). Hence, attrition decreasing measures should be continued in the same intensity for some time after the disappearance of clinical signs of dry eye. The pharmacological guidance by therapy, leading to the reconstitution of the epithelial tissue, needs to be adapted to the individual situation. 

In severe cases of DED, lubrication improvement alone will be insufficient, due to the total dysregulation of normally auto-regulated systems such as normal HA turnover and the self-silencing of minor inflammation by HA, TSG-6 or similar systems. In these cases, neither intensified treatment with immune-modulators, or with antiphlogistic agents, is sufficient. Here, the temporary introduction of a mechano-transduction buffer needs to be considered, to break the vicious circle maintained by tear fluid deficiency and excessive attrition. A properly fitted contact lens (CL), additional to lubricant treatment, could break the circle in these cases. As a temporary layer between the surfaces, careful CL fitting is imperative, resulting in a decrease and enhanced spreading of pressure exerted from the lids to the surfaces of the eye, significantly alleviating attrition. Such use of CLs could deserve more attention in the treatment of DEDs than currently applied [75]. CL wear itself, in turn, is known to increase enzymatic activity at the surface such that as of plasmin [133]. As plasmin can degrade HA, it can break down HMW molecules to small size molecules, changing the characteristics of topical HA from anti-inflammatory friend to a pro-inflammatory foe. The occurrence of inflammatory cells lowers the local pH further, facilitating the degradation of HMW HA to LMW HA. In this environment, activated enzymes, such as plasmin and plasminogen activator, are known to contribute to destruction of cytokines and ECM proteins such as fibronectin, needed for epithelial recovery and healing. On the other hand, whereas low-molecular-size polymers of HA appear to function as endogenous ‘‘danger signals’’, even smaller fragments can again ameliorate these effects [134]. The application of CLs on inflamed surfaces can be tricky and could even consider the use of protein inhibitors [135]—which usually are present in normal tears [136]. The delicacy of CL wear in these cases restricts the time and applicability for this treatment. Hence, the possible side effects of CL wear in these patients need to be carefully evaluated. Different CL materials and coatings must be considered on an individual basis. The success of this therapy is nowadays possible to monitor, even with CL in place, by the use of a high resolution anterior segment optical coherence tomography (OCT), allowing one to judge the increase of epithelial thickness, reversing the reported dry eye associated epithelial thinning [137]. 

The use of pain as a diagnostic criterium can be misleading, due to phenomena known as hyperalgesia and peripheral sensitization [79]. Already, in initial phases of DED, attrition could lead to allodynia [138], describing a nociception in dry eye patients, where they may perceive the normal movement of the lids as irritating, reaching the level of pain. This pain sensation is only partly accessible to treatment, and problematic [139]. Sensations and pain are amongst the first signs of attrition in the pathophysiology of DED. However, the dissociation of signs and symptoms during the disease make them so far unreliable for their use as indicators of improvement. The complete disappearance of neurologic symptoms may follow the epithelial recovery with considerable delay. However, only when neither signs or symptoms are no more perceived and the physiological reconstitution following anatomical recovery is complete, then the situation of complete recovery from DED and the absence of enhanced attrition might have been reached. 

## 3. Conclusions

Attrition, as a mechanical driving force, can contribute to the initiation and maintenance of the vicious circle of DED. Understanding the principle of impaired mechano-transduction in DED and its association with pain could offer a new understanding and insight in ocular pathophysiology and could contribute to the improved treatment of the disease.
